# Supplementary material for: Transcriptomic landscape of the blastema niche in regenerating adult axolotl limbs at single-cell resolution
Source: Nat Commun. 2018 Dec 4;9:5153. doi: 10.1038/s41467-018-07604-0 (PMC6279788; doi:10.1038/s41467-018-07604-0)
Supplement: Supplementary file 1 — Supplementary Information [file 41467_2018_7604_MOESM1_ESM.pdf]

Supplementary information for Leigh et al.

Transcriptomic landscape of the blastema niche in regenerating adult axolotl limbs at single-cell resolution

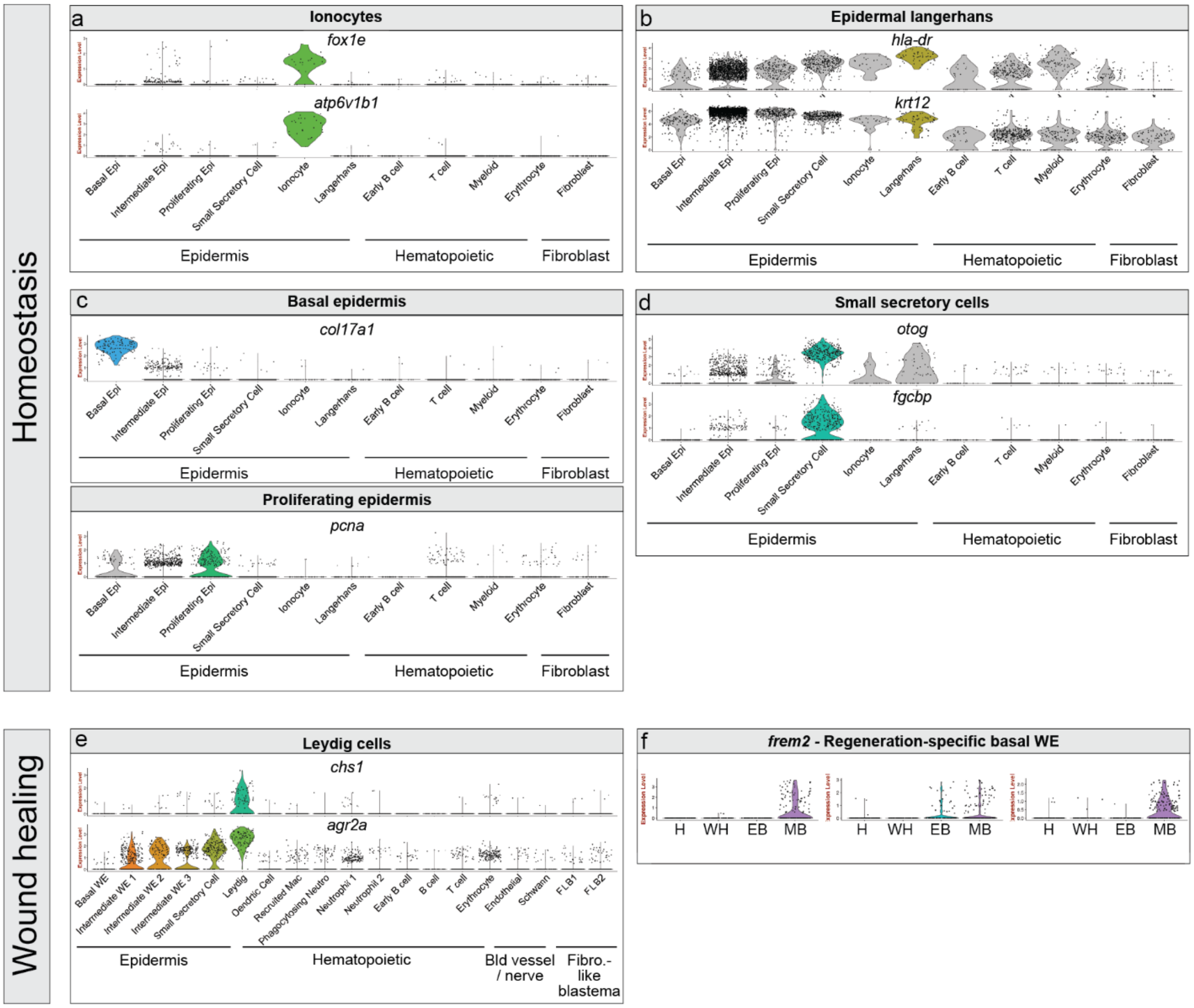

Supplementary Figure 1: Marker genes for epidermal populations. (a) Violin plots depicting ionocyte-enriched genes *fox1e*, and *atp6v1b1* in homeostatic limbs. (b) Violin plots depicting epidermal Langerhans-enriched genes *hla-dr*, and *krt12* in homeostatic limbs. (c) Violin plots depicting basal epidermis-enriched gene *col17a1* and proliferating epidermis enriched-gene *pcna*, in homeostatic limbs. (d) Violin plots depicting small secretory cells-enriched genes *otog* and *fgcbp* in homeostatic limbs. (e) Violin plots depicting Leydig cell-enriched genes *chs1* and *agr2a* at wound healing. (f) Expression of three isoforms of *frem2* at homeostasis (H), wound healing (WH), early-bud blastema (EB) and medium-bud blastema (MB). Each dot represents an individual cell. See Supplementary Table II for population abbreviation definitions.

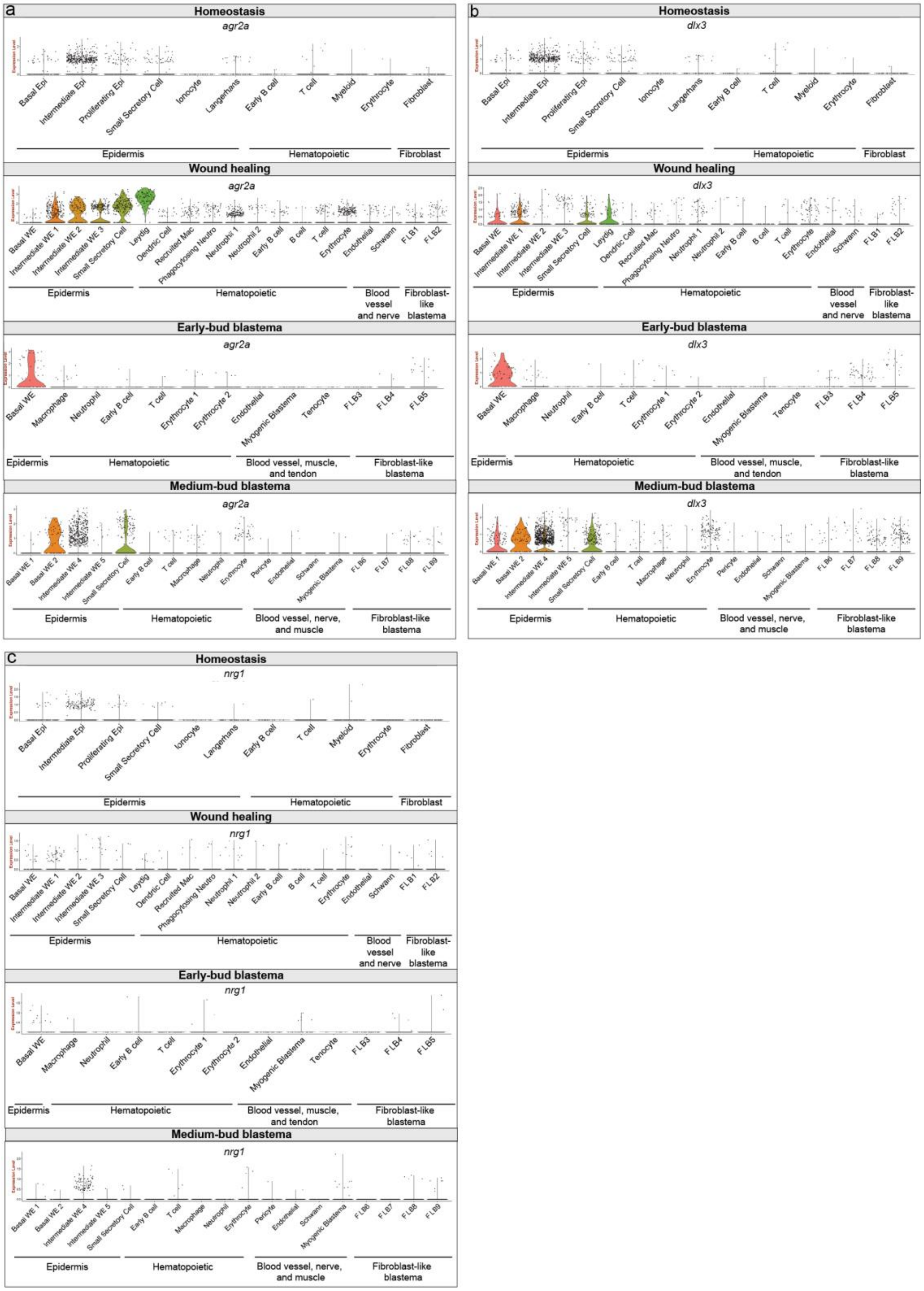

Supplementary Figure 2: Expression of genes previously described to be expressed in or near the wound epidermis. (a-c) Violin plots depicting (a) *agr2a*, (b) *dlx3*, and (c) *nrg1* in homeostatic limbs, wound healing, early-bud blastemas, and medium-bud blastemas. Each dot represents an individual cell. See Supplementary Table II for population abbreviation definitions.

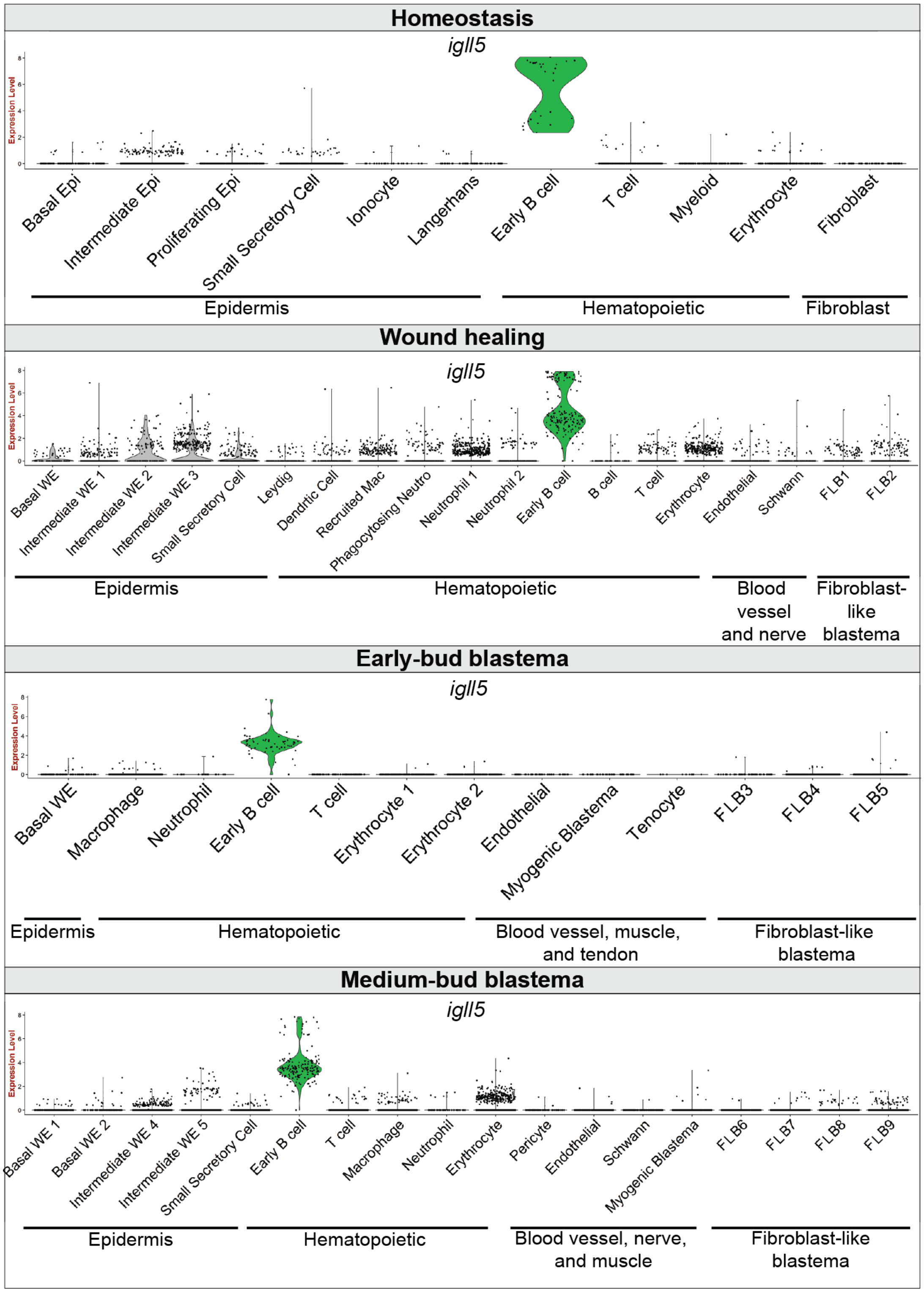

Supplementary Figure 3: Expression of B cell genes *igll5*. Violin plots depicting *igll5* expression in homeostatic limbs, wound healing, early-bud blastemas, and medium-bud blastemas. Each dot represents an individual cell.

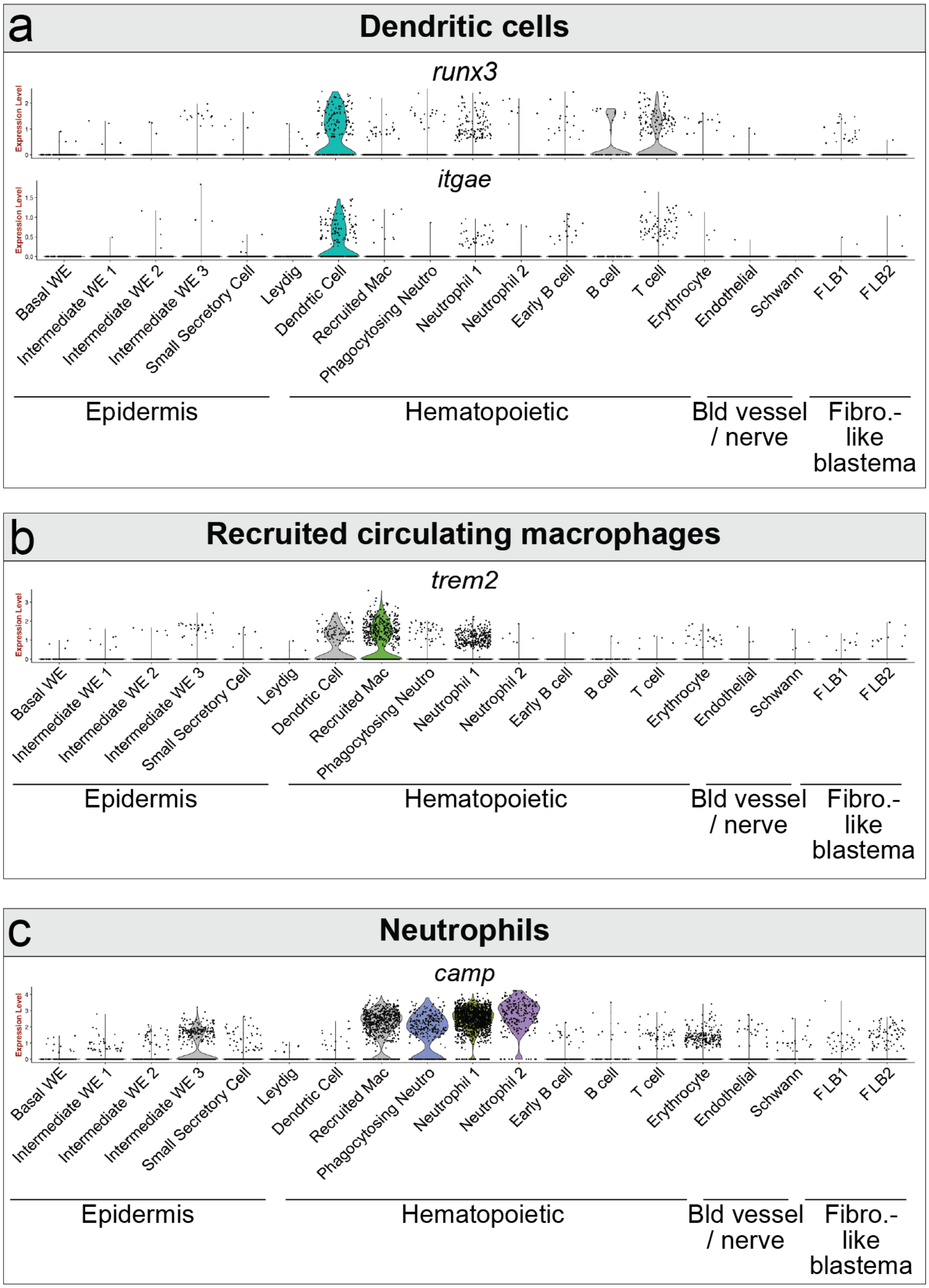

Supplementary Figure 4: Expression of myeloid cell-enriched genes at wound healing (a) Violin plots depicting dendritic cell markers *runx3* and *itgae*. (b) Violin plots depicting recruited macrophage enriched genes *trem2*. (c) Violin plots depicting neutrophil enriched gene *camp*. Each dot represents an individual cell.

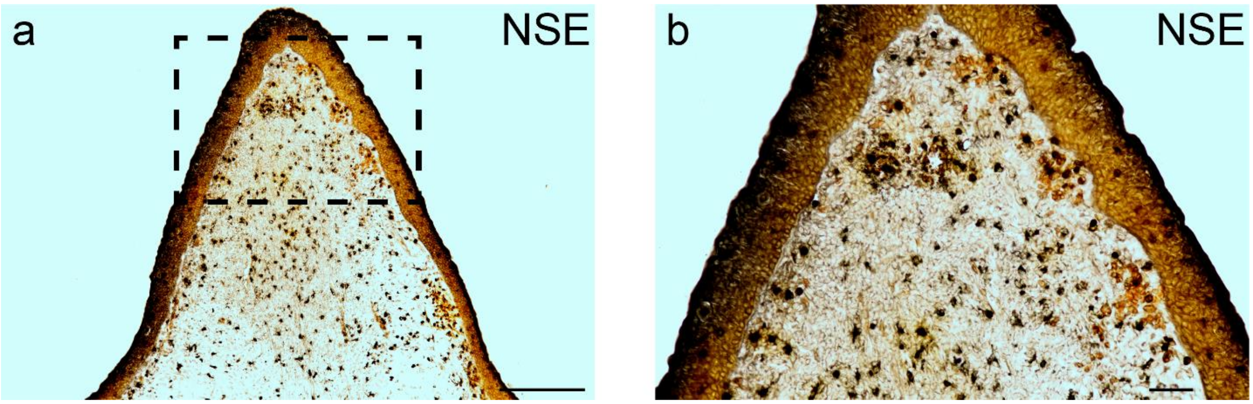

Supplementary Figure 5: Myeloid cells are present in medium-bud blastemas. α-naphthyl acetate (NSE) staining of a medium-bud blastema, (a) 10x magnification (b) area outlined in a at 20x magnification. All scale bars are 100μm. n = 4 medium-bud blastemas from four animals.

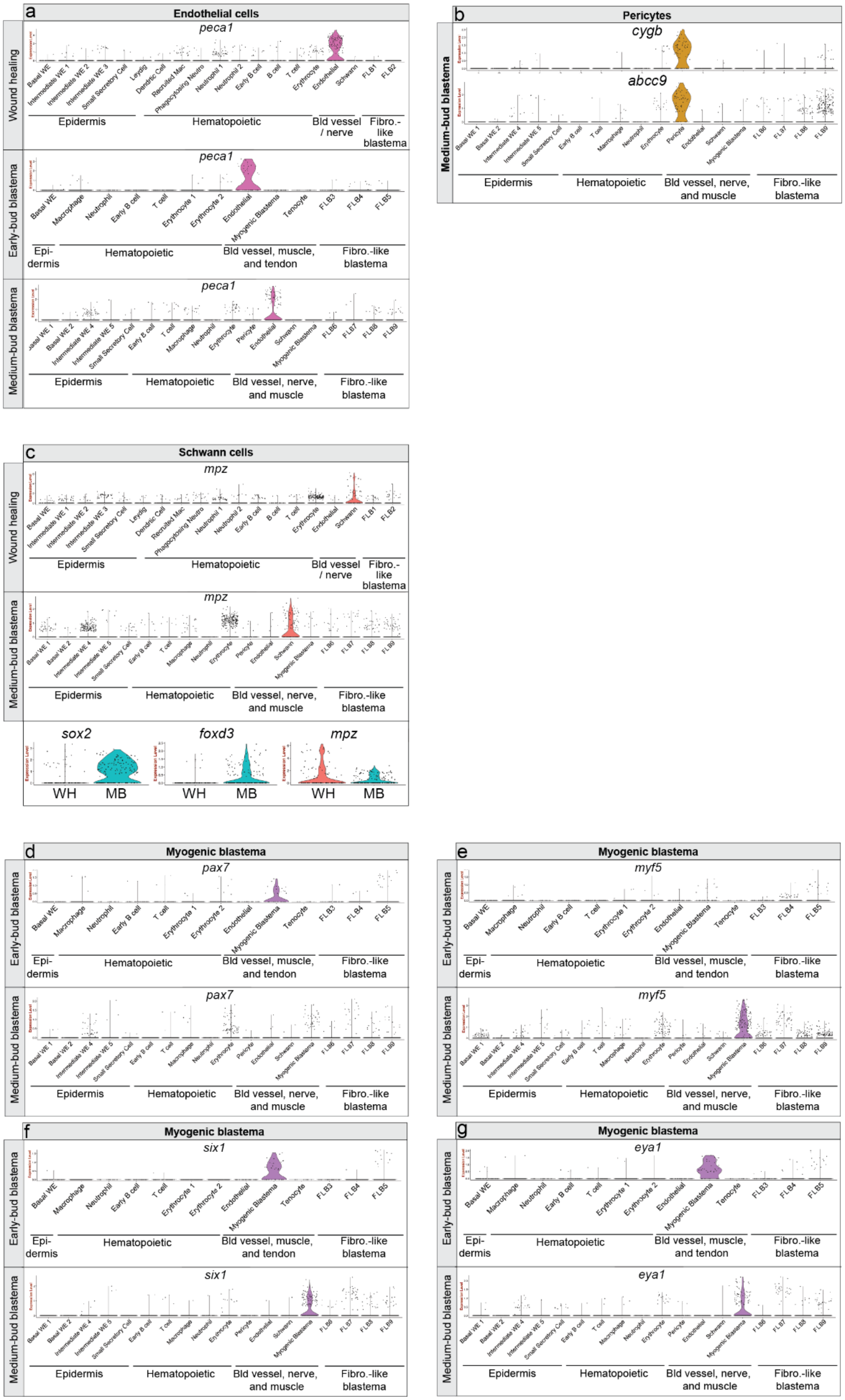

a
Endothelial cells
Wound healing
peca1
Epidermis
Hematopoietic
Bld vessel / nerve
Fibro.-like blastema
Early-bud blastema
peca1
Epi-dermis
Hematopoietic
Bld vessel, muscle, and tendon
Fibro.-like blastema
Medium-bud blastema
peca1
Epidermis
Hematopoietic
Bld vessel, nerve, and muscle
Fibro.-like blastema
b
Pericytes
Medium-bud blastema
cygb
abcc9
Epidermis
Hematopoietic
Bld vessel, nerve, and muscle
Fibro.-like blastema
c
Schwann cells
Wound healing
mpz
Epidermis
Hematopoietic
Bld vessel / nerve
Fibro.-like blastema
Medium-bud blastema
mpz
Epidermis
Hematopoietic
Bld vessel, nerve, and muscle
Fibro.-like blastema
sox2
foxd3
mpz
WH
MB
WH
MB
WH
MB
d
Myogenic blastema
Early-bud blastema
pax7
Epi-dermis
Hematopoietic
Bld vessel, muscle, and tendon
Fibro.-like blastema
Medium-bud blastema
pax7
Epidermis
Hematopoietic
Bld vessel, nerve, and muscle
Fibro.-like blastema
e
Myogenic blastema
Early-bud blastema
myf5
Epi-dermis
Hematopoietic
Bld vessel, muscle, and tendon
Fibro.-like blastema
Medium-bud blastema
myf5
Epidermis
Hematopoietic
Bld vessel, nerve, and muscle
Fibro.-like blastema
f
Myogenic blastema
Early-bud blastema
six1
Epi-dermis
Hematopoietic
Bld vessel, muscle, and tendon
Fibro.-like blastema
Medium-bud blastema
six1
Epidermis
Hematopoietic
Bld vessel, nerve, and muscle
Fibro.-like blastema
g
Myogenic blastema
Early-bud blastema
eya1
Epi-dermis
Hematopoietic
Bld vessel, muscle, and tendon
Fibro.-like blastema
Medium-bud blastema
eya1
Epidermis
Hematopoietic
Bld vessel, nerve, and muscle
Fibro.-like blastema

Supplementary Figure 6: Marker genes for putative blastema populations—endothelial cells, pericytes, Schwann cells, and myogenic blastema. (a) Violin plot depicting endothelial cell marker *pecam1* at wound healing, early-bud blastema, and medium-bud blastema stages. (b) Violin plot depicting pericyte markers *cygb* and *abcc9* in the medium-bud blastema. (c) Violin plot depicting Schwann cell marker *mpz* at wound healing and in the medium-bud blastema (top) and violin plots depicting Schwann cell expression of *sox2*, *foxd3*, and *mpz* at wound healing (WH) and medium-bud blastema (MB) stages. (d-g) Violin plot depicting myogenic blastema markers (d) *pax7*, (e) *myf5*, (f) *six1*, and (g) *eya1* in the early- and medium-bud blastema. Each dot represents an individual cell.

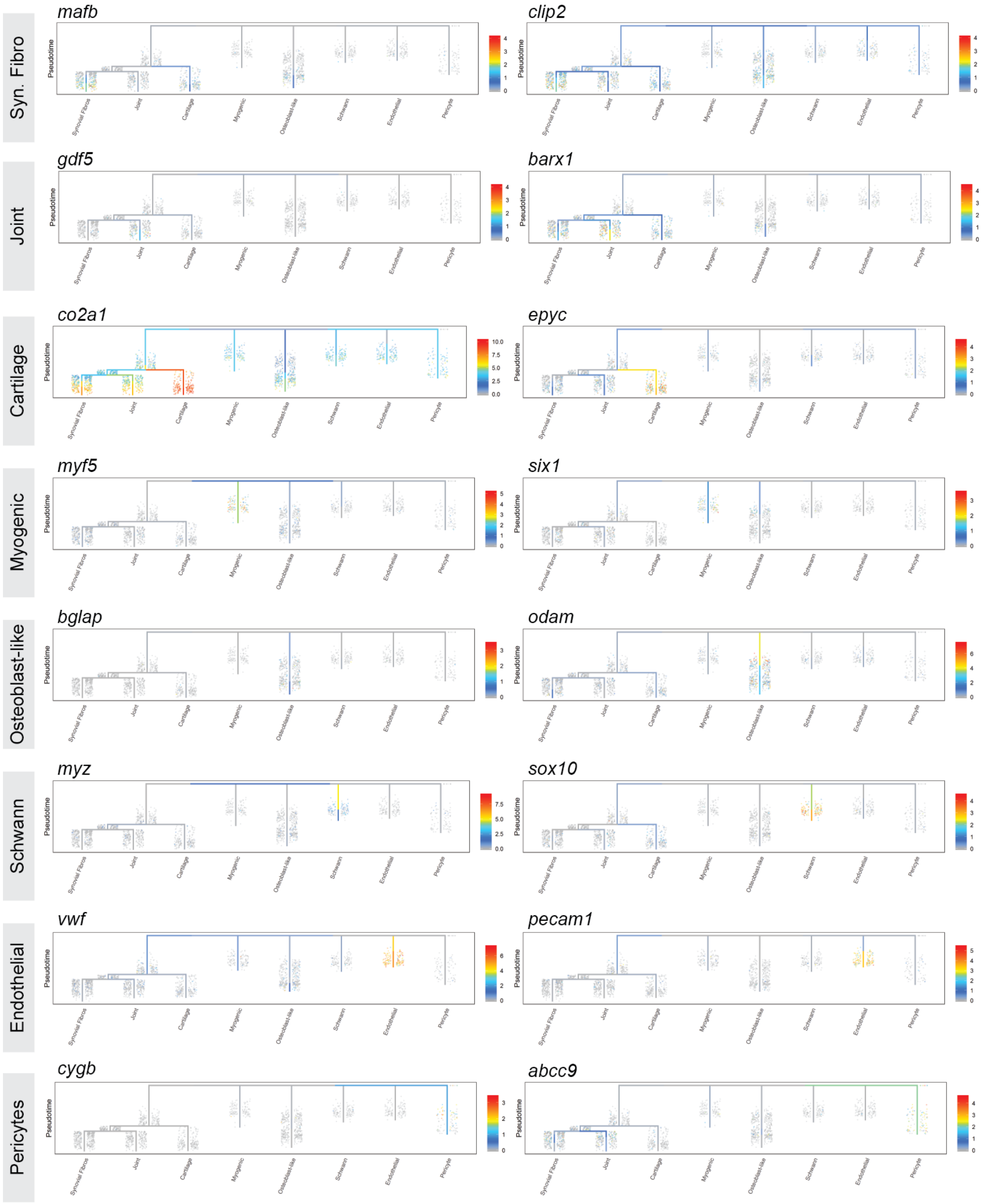

Supplementary Figure 6: Tip markers plotted on trajectory tree. Population-enriched genes overlaid on the trajectory tree. Markers for synovial fibroblasts (*mafb*, *clip2*), joint-like (*gdf5*, *barx1*), cartilage (*col2a1*, *epyc*), myogenic blastema (*myf5*, *six1*), osteoblast-like fibroblasts (*bglap*, *odam*), Schwann cells (*myz*, *sox10*), endothelial cells (*vwf*, *pecam1*), and pericytes (*cygb*, *abcc9*).

Supplementary Table 1: Sample metadata and data accessibility

Sample Name	DPA	Stage	Replicate	Sex	Length (cm)	Side	Cells	GEO Accession #
S1	23	Medium-bud	1	M	21.5	R	1679	GSE121737
S2	23	Medium-bud	2	F	18.25	R	1836	GSE121737
S4	23	Medium-bud	3	M	21	L	3212	GSE121737
N1	23	Medium-bud	4	F	19.5	R	2188	GSE121737
N2	23	Medium-bud	5	F	17.5	L	2260	GSE121737
N3	23	Medium-bud	6	M	19	L	1356	GSE121737
S3	14	Early-bud	1	F	18.5	L	1291	GSE121737
S5	14	Early-bud	2	M	21.25	R	722	GSE121737
N4	3	Wound healing	1	F	17	R	2678	GSE121737
N5	3	Wound healing	2	M	21.5	L	1986	GSE121737
N6	3	Wound healing	3	M	20	R	2606	GSE121737
S1	N/A	Homeostasis	1	M	19	R	981	GSE121737
S2	N/A	Homeostasis	2	M	18	L	2440	GSE121737

Supplementary Table 2: Primers used to clone RNA *in situ* hybridization probes

**Gene**	**Primer Name**	**Sequence**
krt17	krt17 F	CCTCTTGGACGTGAAGACC
krt17	krt17 R	CCAGAGAAGATGAGCATACATCGG
frem2	frem2 F	GTGCCCAGACGTGCCTTATTTC
frem2	frem2 R	GGCAACGCCTTTCATAATACTTGCC
apoeb	apoeb F	GGACAAGGTGAAGGAGAAGATG
apoeb	apoeb R	AGGTGGAAGGAGGAGCGATAG
otog	otog F	CATTATGGCACTGTTCATCGC
otog	otog R	GAGTCTTCAACCTTTGTGGAA
krt12	krt12 F	GACAACGTACACAGCCACG
krt12	krt12 R	CCAGTCTTTGAATGCCTTGC
trac	trac F	AGGACAGCCTTTGGTCACAGTG
trac	trac R	GCTGCCCATTGCCATTATTG
lum	lum F	CTGCTTAGGAATAAGAGCTTTCGG
lum	lum R	AAGCTTAGGAATACTCTGAGATGCTTC
dpt	dpt F	GGGACAGAATGGTATCAGAC
dpt	dpt R	CTCGCATTGATTGGTTGCAG
