# Supplementary material for: Transcriptomic landscape of the blastema niche in regenerating adult axolotl limbs at single-cell resolution
Source: Nat Commun. 2018 Dec 4;9:5153. doi: 10.1038/s41467-018-07604-0 (PMC6279788; doi:10.1038/s41467-018-07604-0)
Supplement: Supplementary file 18 — Description of Additional Supplementary Files [file 41467_2018_7604_MOESM18_ESM.docx]

**Title:** Supplementary Data 1:
**Description:** Populations and their abbreviations identified across single-cell RNAseq analysis. Clusters were determined by unbiased clustering in Seurat followed by evaluation of marker genes to determine population identity.

**Title:** Supplementary Data 2:
**Description:** Population markers for cells collected at homeostasis. Marker genes as determined by Seurat for each population at homeostasis are shown.

**Title:** Supplementary Data 3:
**Description:** Population markers for cells collected at wound healing. Marker genes as determined by Seurat for each population at wound healing are shown.

**Title:** Supplementary Data 4:
**Description:** Population markers for cells collected at early-bud blastema. Marker genes as determined by Seurat for each population at early-bud blastema are shown.

**Title:** Supplementary Data 5:
**Description:** Population markers for cells collected at medium-bud blastema. Marker genes as determined by Seurat for each population at medium-bud blastema are shown.

**Title:** Supplementary Data 6:
**Description:** Undifferentiated distal blastema markers defined by URD at medium-bud blastema stage. Non-immune blastema cells from the medium-bud blastema were re-clustered and populations within this one time point were identified. Marker genes as determined by URD using AUCPR to identify enriched genes in distal undifferentiated blastema cells are shown.

**Title:** Supplementary Data 7:
**Description:** Osteoblast-like fibroblast markers defined by URD at medium-bud blastema stage. Non-immune blastema cells from the medium-bud blastema were re-clustered and populations within this one time point were identified. Marker genes as determined by URD using AUCPR to identify enriched genes in osteoblast-like fibroblasts are shown.

**Title:** Supplementary Data 8:
**Description:** Synovial fibroblast markers defined by URD at medium-bud blastema stage. Non-immune blastema cells from the medium-bud blastema were re-clustered and populations within this one time point were identified. Marker genes as determined by URD using AUCPR to identify enriched genes in synovial fibroblasts are shown.

**Title:** Supplementary Data 9:
**Description:** Pericyte markers defined by URD at medium-bud blastema stage. Non-immune blastema cells from the medium-bud blastema were re-clustered and populations within this one time point were identified. Marker genes as determined by URD using AUCPR to identify enriched genes in pericytes are shown.

**Title:** Supplementary Data 10:
**Description:** Myogenic blastema markers defined by URD at medium-bud blastema stage. Non-immune blastema cells from the medium-bud blastema were re-clustered and populations within this one time point were identified. Marker genes as determined by URD using AUCPR to identify enriched genes in myogenic blastema cells are shown.

**Title:** Supplementary Data 11:
**Description:** Cartilage-like markers defined by URD at medium-bud blastema stage. Nonimmune blastema cells from the medium-bud blastema were re-clustered and populations within this one time point were identified. Marker genes as determined by URD using AUCPR to identify enriched genes in cartilage-like cells are shown.

**Title:** Supplementary Data 12:
**Description:** Endothelial cell markers defined by URD at medium-bud blastema stage. Nonimmune blastema cells from the medium-bud blastema were re-clustered and populations within this one time point were identified. Marker genes as determined by URD using AUCPR to identify enriched genes in endothelial cells are shown.

**Title:** Supplementary Data 13:
**Description:** Schwann cell markers defined by URD at medium-bud blastema stage. Nonimmune blastema cells from the medium-bud blastema were re-clustered and populations within this one time point were identified. Marker genes as determined by URD using AUCPR to identify enriched genes in Schwann cells are shown.

**Title:** Supplementary Data 14:
**Description:** Joint-like markers defined by URD at medium-bud blastema stage. Nonimmune blastema cells from the medium-bud blastema were re-clustered and populations within this one time point were identified. Marker genes as determined by URD using AUCPR to identify enriched genes in joint-like cells are shown.

**Title:** Supplementary Data 15:
**Description:** Fibro-adipogenic progneitor markers defined by URD at medium-bud blastema stage. Non-immune blastema cells from the medium-bud blastema were re-clustered and populations within this one time point were identified. Marker genes as determined by URD using AUCPR to identify enriched genes in fibro-adipogenic progneitor cells are shown.

**Title:** Supplementary Code:
**Description:** This code contains all R code used in this manuscript including Seurat clustering, URD trajectory analysis, and Monocle pseudotime analysis of the epidermis. This code can be used with the cell by gene matrices found https://www.ncbi.nlm.nih.gov/geo/query/acc.cgi?acc=GSE121737.
