# Supplementary material for: Transcriptomic landscape of the blastema niche in regenerating adult axolotl limbs at single-cell resolution
Source: Nat Commun. 2018 Dec 4;9:5153. doi: 10.1038/s41467-018-07604-0 (PMC6279788; doi:10.1038/s41467-018-07604-0)
Supplement: Supplementary file 19 — Reporting Summary [file 41467_2018_7604_MOESM19_ESM.pdf]

nature research

Corresponding author(s): Dr. Jessica Whited

Life Sciences Reporting Summary

Nature Research wishes to improve the reproducibility of the work that we publish. This form is intended for publication with all accepted life science papers and provides structure for consistency and transparency in reporting. Every life science submission will use this form; some list items might not apply to an individual manuscript, but all fields must be completed for clarity.

For further information on the points included in this form, see Reporting Life Sciences Research. For further information on Nature Research policies, including our data availability policy, see Authors & Referees and the Editorial Policy Checklist.

Please do not complete any field with "not applicable" or n/a. Refer to the help text for what text to use if an item is not relevant to your study. For final submission: please carefully check your responses for accuracy; you will not be able to make changes later.

Experimental design

**1. Sample size**<br>Describe how sample size was determined.	No statistical methods were used to determine sample size. Two to six biological replicates were performed for single-cell RNA-seq as we typically find to be adequate for reproducible results using bulk RNA sequencing and RNA in situ hybridizations.
**2. Data exclusions**<br>Describe any data exclusions.	No data were excluded.
**3. Replication**<br>Describe the measures taken to verify the reproducibility of the experimental findings.	All in situs were performed on more than three biological and experimental replicates. Single-cell data align with bulk transcriptome sequencing and other single-cell data for a different manuscript generated by a different protocol in our lab. Single-cell capture was performed on three individual days.
**4. Randomization**<br>Describe how samples/organisms/participants were allocated into experimental groups.	All cell clustering was performed by unbiased statistical methods.
**5. Blinding**<br>Describe whether the investigators were blinded to group allocation during data collection and/or analysis.	Blinding was not relevant because cell clustering was performed using an unbiased pipeline.

Note: all in vivo studies must report how sample size was determined and whether blinding and randomization were used.

6. Statistical parameters

For all figures and tables that use statistical methods, confirm that the following items are present in relevant figure legends (or in the Methods section if additional space is needed).

n/a	Confirmed	
☐	☒	The exact sample size (*n*) for each experimental group/condition, given as a discrete number and unit of measurement (animals, litters, cultures, etc.)
☐	☒	A description of how samples were collected, noting whether measurements were taken from distinct samples or whether the same sample was measured repeatedly
☐	☒	A statement indicating how many times each experiment was replicated
☐	☒	The statistical test(s) used and whether they are one- or two-sided<br>*Only common tests should be described solely by name; describe more complex techniques in the Methods section.*
☐	☒	A description of any assumptions or corrections, such as an adjustment for multiple comparisons
☐	☒	Test values indicating whether an effect is present<br>*Provide confidence intervals or give results of significance tests (e.g. P values) as exact values whenever appropriate and with effect sizes noted.*
☒	☐	A clear description of statistics including central tendency (e.g. median, mean) and variation (e.g. standard deviation, interquartile range)
☐	☒	Clearly defined error bars in all relevant figure captions (with explicit mention of central tendency and variation)

See the web collection on statistics for biologists for further resources and guidance.

Software

Policy information about availability of computer code

7. Software

Describe the software used to analyze the data in this study.	Seurat v2.3.4, R 3.4.1, Bowtie v1.2.2, Python 2.7, samtools 1.3.1, pysam 0.9.1, Monocle v2.6.4, bcl2fastq v2.18. For URD we used R 3.5.1, URD v1.0.1.

For manuscripts utilizing custom algorithms or software that are central to the paper but not yet described in the published literature, software must be made available to editors and reviewers upon request. We strongly encourage code deposition in a community repository (e.g. GitHub). *Nature Methods* guidance for providing algorithms and software for publication provides further information on this topic.

Materials and reagents

Policy information about availability of materials

8. Materials availability

Indicate whether there are restrictions on availability of unique materials or if these materials are only available for distribution by a third party.	No unique materials were used.

9. Antibodies

Describe the antibodies used and how they were validated for use in the system under study (i.e. assay and species).	No antibodies were used.

10. Eukaryotic cell lines

a. State the source of each eukaryotic cell line used.	No eukaryotic cell lines were used
b. Describe the method of cell line authentication used.	No eukaryotic cell lines were used
c. Report whether the cell lines were tested for mycoplasma contamination.	No eukaryotic cell lines were used
d. If any of the cell lines used are listed in the database of commonly misidentified cell lines maintained by ICLAC, provide a scientific rationale for their use.	No eukaryotic cell lines were used

Animals and human research participants

Policy information about studies involving animals; when reporting animal research, follow the ARRIVE guidelines

11. Description of research animals

Provide all relevant details on animals and/or animal-derived materials used in the study.	Ambystoma mexicanum, strain white (d/d), for single-cell RNAseq: females and males 1+ year of age and snout to tail length of 17-21.5cm. For RNA in situ hybridizations males and females 6+ months of age and snout to tail length of 9.5cm - 22cm.

Policy information about studies involving human research participants

12. Description of human research participants

Describe the covariate-relevant population characteristics of the human research participants.	*Provide all relevant information on human research participants, such as age, gender, genotypic information, past and current diagnosis and treatment categories, etc. OR state that the study did not involve human research participants.*
